# KYNU in macrophages contributes to the unique immune feature of LUAD via integrating single-cell and bulk RNA sequencing data: an exploratory analysis

**DOI:** 10.1371/journal.pone.0351622

**Published:** 2026-06-12

**Authors:** Jie Yao, Changshuai Zhou, Liren Ding

**Affiliations:** 1 Department of Thoracic Surgery, The Second Affiliated Hospital Zhejiang University School of Medicine, Hangzhou, China; 2 Department of Cardiothoracic, The Fourth Affiliated Hospital Zhejiang University School of Medicine, Yiwu, China; 3 Department of Respiratory Medicine, The Second Affiliated Hospital Zhejiang University School of Medicine, Hangzhou, China; Xiangya Hospital Central South University, CHINA

## Abstract

**Background:**

Lung adenocarcinoma (LUAD) is a predominant subtype of lung cancer associated with an unfavorable prognosis. However, the roles of the tumor microenvironment (TME) and Kynureninase (KYNU) in LUAD remain largely unclear. This study aimed to investigate the potential role of KYNU in macrophages and LUAD.

**Methods:**

All LUAD related data were downloaded from The Cancer Genome Atlas (TCGA) and Gene Expression Omnibus (GEO) databases. The expression of KYNU was analyzed across different cell types following dimensionality reduction analysis. Immune cell infiltration and immunotherapy response prediction were performed using CIBERSORT and TIMER, respectively. Gene set variation analysis (GSVA) was employed for functional enrichment.

**Results:**

Among all immune cells in LUAD, KYNU was primarily expressed in monocytes and macrophages. The upregulated genes in KYNU+macrophages group were significantly enriched in in gene ontology (GO) terms related to antigen processing and presentation. There were increased MHC-I/ MHC-II signal interactions between KYNU+macrophages and B cells as well as T cells. In LUAD patients with higher proportions of KYNU+macrophages, a significantly greater number of patients benefited from immunotherapy (*p* = 0.033). GSVA results indicated that the MHC pathway was significantly activated in high KYNU+macrophage group.

**Conclusions:**

KYNU is primarily in LUAD macrophages, contributing to the distinct immune features and correlating with the enhanced antigen presentation in LUAD. This study preliminarily confirms that KYNU may serve as a potential biomarker for immunotherapy.

## Introduction

As an important leading cause of cancer mortalities, lung cancer accounts for more than 20% cancer related deaths around world [1]. There are two main subtypes in lung cancer, comprising small cell lung cancer and non-small cell lung cancer (NSCLC) [2]. Of which, approximately 85% of the lung cancer cases belong to NSCLC [3]. As a predominant subtype of lung cancer, NSCLC has been histologically clustered into lung adenocarcinoma (LUAD), lung squamous cell carcinoma, and lung large-cell carcinoma, and LUAD is the most common subtype, reaching about 40% of all lung cancers [4]. It has been widely documented that LUAD often exhibits high tumor heterogeneity and distinct cellular features, thereby significantly affecting the tumor progression and patients’ responses to various therapies [5]. Accordingly, despite significant advances in chemotherapy, tyrosine kinase inhibitor (TKI) therapy, and immunotherapy, the 5‑year overall survival rate for LUAD patients remains unsatisfactory, at approximately 15% [6–8]. Currently, several immunotherapy checkpoint inhibitors, for example inhibitors of the PD-1 axis, have been reported to be applied for the first-line treatment of advanced NSCLC patients, whereas only about 60% patients exhibit effective responses to immunotherapy [3,9]. In LUAD, the deepening details in tumor immune microenvironment (TIME) remain largely unknown, which continues to be a critical obstacle to advancing treatment and improving prognosis for patients. Therefore, it is imperative to further understand the TIME and explore vital targets in LUAD, in order to provide more reference information for the clinical management of LUAD patients.

As a complex system, TIME comprises multiple immune cells with heterogeneity, in which all members exhibit key impacts on the therapy responses and prognosis of LUAD patients [3]. Among which, macrophages are the predominantly abundant immune cells infiltrating in tumor microenvironment (TME) [10]. Macrophages could be polarized into different phenotypes based on the diverse signals, including classically (M1 macrophages) and alternatively (M2 macrophages) activated cells [11]. Typically, the M1 macrophages exert the pro-inflammatory and antitumor roles, while M2 macrophages display a negative role in tumors via suppressing adaptive immunity [11]. In LUAD patients, high infiltration of M2 macrophages has been indicated to correlate with the inferior prognosis [12]. Xu et al. have documented that modulating macrophage polarization can affect lung cancer progression and the efficacy of immunotherapy [13]. Nevertheless, more deepening details about macrophages in LUAD remain largely unclear.

Kynureninase (KYNU), as a member in Tryptophan metabolism, exerts important role in the synthesis of NAD+ cofactors, functioning as a hydrolase through Kynurenine pathway [14]. Moreover, there is KYNU expression in almost all organs, especially higher expression in liver, the urinary bladder and the appendix [15]. Accumulating evidences have indicated that KYNU participates in not only various inflammatory diseases but also various cancers, like breast cancer and cutaneous squamous cell carcinoma [16,17]. Furthermore, KYNU is closely associated with the remodeling of the immune microenvironment. Studies have found that KYNU, as a biomarker for tumor‑associated macrophages, could reduce kynurenine accumulation and ultimately reprogram tumor‑associated macrophage (TAM) polarization, thereby enhancing anti‑tumor immunity [18]. Yang et al. [19] demonstrated that CAR‑T cells overexpressing KYNU enhance their cancer‑killing capacity by degrading kynurenine within the immunosuppressive tumor microenvironment. However, potential role of KYNU in LUAD has been rarely explored, especially involving its role in the TME of LUAD.

Hence, in this present work, we aimed to investigate the crucial role of KYNU in the TIME of LUAD, integrating single-cell RNA sequencing data and bulk RNA sequencing data from multiple databases. Our findings are expected to give more insights into the understanding of TME in LUAD, especially regarding the potential of KYNU in LUAD.

## Materials and methods

### Data resources

All LUAD related data were collected from The Cancer Genome Atlas (TCGA, https://portal.gdc.cancer.gov/projects/TCGA-LUAD) and Gene Expression Omnibus (GEO, http://www.ncbi.nlm.nih.gov/geo/) databases.

Totally 490 LUAD patients’ mRNA expression data and the corresponding clinical information were downloaded from the TCGA database. Moreover, three datasets, GSE117570, GSE135222 and GSE123902, were obtained from GEO database. The single cell RNA-seq data of 3 LUAD samples were downloaded from GSE117570 (excluding 4 adjacent normal samples and a lung squamous cell carcinoma sample, which were ineligible for this study), and 7 untreated early-stage LUAD samples were downloaded from GSE123902. In GSE135222 dataset, there were a total of 27 advanced LUAD patients receiving anti-PD-1/PD-L1 treatment, and the corresponding expression data and clinical information were downloaded. All the patients from this public data have written the informed consent, and their records cannot be identified in this study.

### Single cell RNA sequencing data analysis

Firstly, the raw sequencing reads were aligned to the GRCh38 human reference genome. Feature-barcode matrices were generated using the Cell Ranger with the standard pipeline. Cells meeting the following criteria were included in the study: (1) gene numbers between 200 and 20000; (2) unique molecular identifier (UMI) count > 300; (3) mitochondrial gene percentage < 40%. After screening the top 2000 highly variable genes (HVGs) from the normalized matrix, the principal component analysis (PCA) was used for the dimensionality reduction. The Louvain clustering algorithm from Seurat was then employed for clustering analysis [20]. The top 30 PCs were selected for uniform manifold approximation and projection (UMAP) to visualize the cell clustering results [21]. Cell types were annotated by known cell markers and SingleR package [20].

For a systematic analysis of cell-cell interaction, cellular communication analysis was performed utilizing Cellchat with default parameters [22].

### Cell–cell communication analysis

To investigate potential interactions among different cell types, we employed the CellChat (v2.1.2) algorithm. The input for this algorithm was a merged Seurat object containing macrophages and other cell types within the TME. After creating the CellChat object, we constructed a reference database based on secretory signaling pathways. We inferred specific receptor–ligand interactions and communication probabilities between different cell types using the computeCommunProb and computeCommunProbPathway functions, respectively.

### Differentially expressed gene analysis

Differentially expressed genes (DEGs) between KYNU+ and KYNU- macrophages were calculated by FindMarkers, based on logFC.threshold = 0.25, test.use = “wilcox”, min.pct = 0.1, and *p* < 0.05. The clusterProfiler R package was used to conduct GO enrichment analysis, and the GO terms with *p* < 0.05 were considered significantly enriched.

### Immune cell infiltration and immunotherapy response prediction

The CIBERSORT (Cell-type Identification by Estimating Relative Subsets of RNA Transcripts) and TIMER (Tumor Immune Estimation Resource, https://cistrome.shinyapps.io/timer/) algorithms were employed to assess the correlation between the KYNU gene and immune cells.

Tumor immune dysfunction and exclusion (TIDE) analysis was then conducted to predict LUAD patients’ immune response to immunotherapy. Moreover, the signature gene set involved in the seven-step cancer-immunity cycle was identified based on the tumor immunophenotype (TIP) website (http://biocc.hrbmu.edu.cn/TIP/index.jsp), which was used as the input data to estimate the immune activities of different groups.

### Survival analysis

The R survival package and survminer package (https://CRAN.R-project.org/package=survminer) were used to estimate the overall survival of various groups, based on Kaplan-Meier method. The log-rank was used to test the significance of difference. *P* < 0.05 was considered statistically significant.

### Functional enrichment analysis

Enrichment analysis of DEGs was performed using the “clusterProfiler” R package. To collect more functional information, Gene set variation analysis (GSVA) was then conducted. Basing on MSigDB signature gene sets “C2 Canonical pathways” (https://www.gseamsigdb.org/gsea/msigdb/index.jsp), GSVA algorithm was used to calculate the pathway scores. FDR.cut = 0.05 and logFC.cut = 0.5 were considered significant threshold values.

### Statistical analysis

To evaluate the correlation between hub group and overall survival, the Cox regression analysis was carried out to determine confidence interval (CI) of different cutoffs. The correlation among different groups was determined by Pearson correlation analysis. The significance of the difference between KYNU- and KYNU+ macrophages was determined using Fisher’s exact test. The comparison between the two groups was performed using the Wilcoxon signed-rank test. All statistical computations were conducted using R software (version 4.0.5), and statistically significant threshold was *p* < 0.05.

## Results

### The expression pattern of KYNU in LUAD samples at a single cell resolution

**Fig 1:**
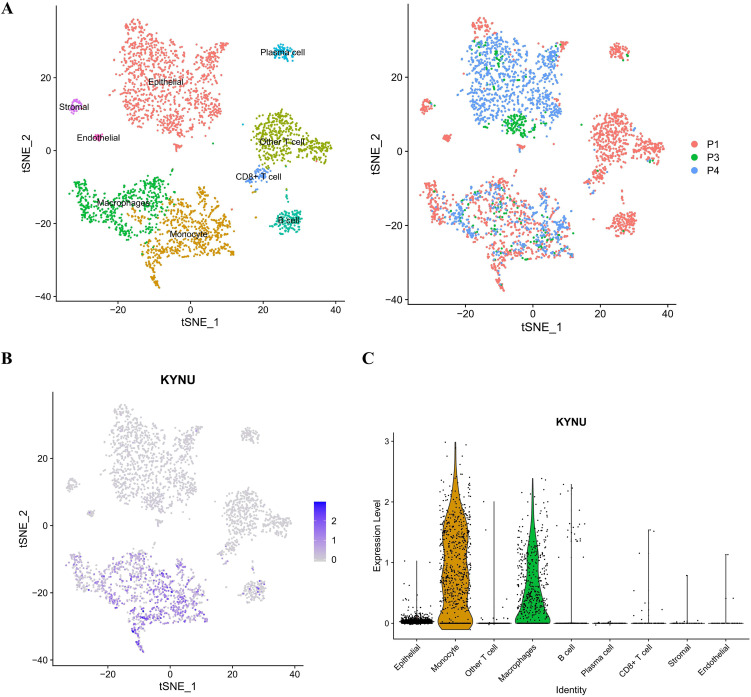
The expression pattern of KYNU in LUAD samples at a single cell resolution. **(A)** All 9 cell clusters annotated in GSE117570. **(B-C)** KYNU was mainly expressed in Monocytes and Macrophages in GSE117570.

To explore the expression pattern of KYNU in LUAD at a single cell resolution, LUAD single cell RNA sequencing data were downloaded. After data normalization and principal component analysis, a total of 3,539 cells were clustered into 9 cell clusters based on 8013 marker genes (Fig 1A). Then, the expression of KYNU was analyzed in all cells, and our data indicated that KYNU was mainly expressed in Monocytes and Macrophages (Fig 1B, 1C). Considering the complex and crucial role of macrophages in LUAD, the expression of KYNU in macrophages was predominantly studied in our present work. Subsequently, we validated the findings using single‑cell RNA sequencing data from seven untreated and relatively early‑stage lung adenocarcinoma samples in the GSE123902 database. The results showed that KYNU is primarily expressed in monocytes and macrophages. In the analysis of these seven samples, we also identified clustered dendritic cells and found that KYNU is expressed in dendritic cells as well (S1 Fig A-C). These results are consistent with those obtained from the GSE117570 database.

### KYNU expression was correlated with macrophages and enhanced antigen presentation in LUAD

**Fig 2:**
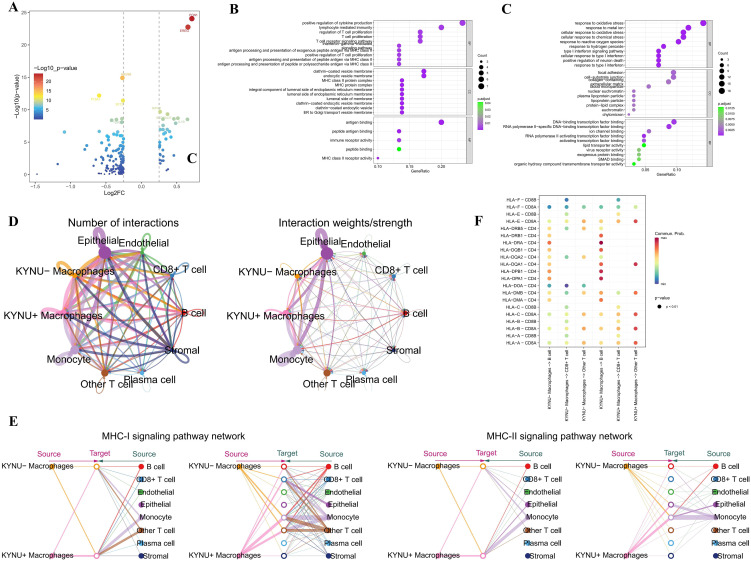
KYNU expression was correlated with macrophages and enhanced antigen presentation in LUAD. **(A)** The differentially expressed genes between KYNU+ macrophages group and KYNU-macrophages group. **(B-C)** The GO enrichment results of upregulated genes in KYNU+ macrophages group (B) and KYNU-macrophages group **(C)**, respectively. **(D)** The cell communications among KYNU+ macrophages, KYNU-macrophages, and other cells. **(E)** The interaction network involving MHC-I signaling pathway and MHC-II signaling pathway between KYNU+macrophages and KYNU-macrophages. **(F)** Correlation between MHC-I/ MHC-II molecules and KYNU+ macrophages, KYNU-macrophages.

Subsequently, based on the KYNU expression status in LUAD macrophages, all samples were then divided into two groups, including KYNU expressed LUAD macrophages (KYNU+macrophages) and KYNU non-expressed LUAD macrophages (KYNU-macrophages). To further explore the differences between the above two groups, the differentially expressed gene analysis was conducted. Compared with KYNU-macrophages group, totally 179 differentially expressed genes (DEGs) were identified in KYNU+macrophages group, comprising 32 upregulated genes and 147 downregulated genes (Fig 2A). Moreover, the GO functional enrichment analysis was performed on the 179 DEGs, in order to get the distinct functional information between KYNU+macrophages group and KYNU-macrophages group. Our results indicated that 32 upregulated genes in KYNU+macrophages group were predominantly significantly enriched in cytokine production, T cell proliferation, and antigen processing and presentation related GO terms (Fig 2B). The 147 upregulated genes in KYNU-macrophages group were mainly significantly enriched in oxidative stress and transcription factor binding related GO terms (Fig 2C). Functional enrichment results implied that KYNU related roles in LUAD macrophages were probably involved in the antigen processing and presentation.

Furthermore, the cell communications among KYNU+macrophages, KYNU-macrophages, and other cells have been analyzed, and the results suggested that more interactions were observed between KYNU+macrophages and other cells (Fig 2D). Besides, basing on the functional enrichment data, the interaction network involving Major Histocompatibility Complex --class I (MHC-I) signaling pathway and MHC-II signaling pathway between KYNU+macrophages and KYNU-macrophages was analyzed. We noticed that there were more MHC-I/ MHC-II signal interactions between KYNU+macrophages and B cells, T cells (Fig 2E). KYNU+macrophages and KYNU-macrophages mainly interacted with B cells and T cells, which was probably realized via MHC-I/ MHC-II molecules as well as their ligand-receptor interactions with CD4, CD8A, CD8B (Fig 2F). Furthermore, the data from the validation set of the GSE123902 database align with the analysis results from the GSE117570 dataset, confirming that KYNU expression is associated with macrophages and enhanced antigen presentation in lung adenocarcinoma (S2 Fig A-C). Thus, our data indicated that KYNU expression was not only correlated with macrophages but also correlated with the enhanced antigen presentation in LUAD.

### The potential clinical value of KYNU in immunotherapy responses of LUAD patients

**Fig 3:**
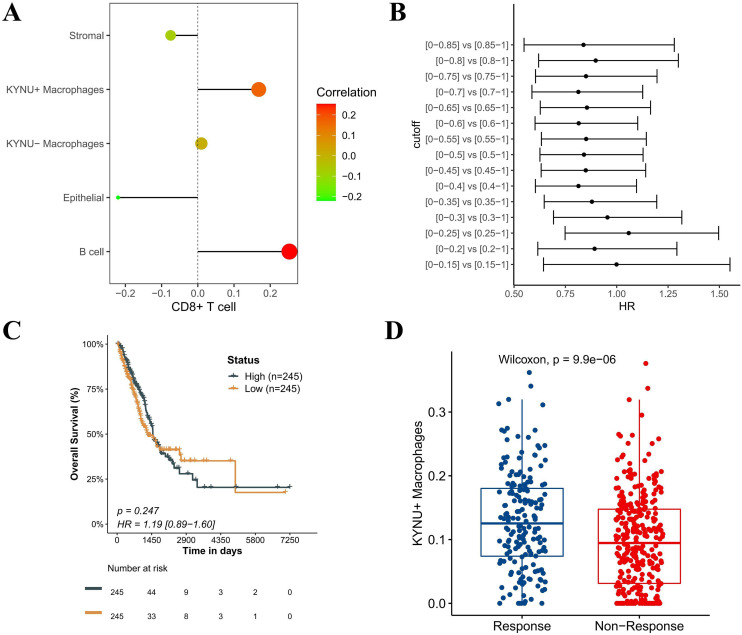
Clinical value of KYNU in immunotherapy responses of LUAD patients basing on TCGA cohort. **(A)** The correlation analysis between CD8 + T cells and other cells. **(B)** The prognostic value of KYNU+ macrophage under various cutoff values. **(C)** Kaplan-Meier curve based on high and low KYNU expression LUAD groups. **(D)** The proportions of KYNU+ macrophages in Responses and Non-Response LUAD patients (using data in GSE135222).

Considering the important role of KYNU in macrophages and LUAD TME, the potential clinical value of KYNU was then analyzed in TCGA-LUAD cohort (bulk RNA sequencing data). Firstly, the correlation analysis between CD8 + T cells and other cells was performed, and we found that there was a strongest correlation between CD8 + T cells and B cells. Moreover, the KYNU+macrophages showed the second highest correlation with CD8 + T cells, while the correlation between KYNU-macrophages and the CD8 + T cells was the weakest (Fig 3A). The correlation between KYNU expression and several crucial immune cells’ infiltration was also analyzed, while we found a relative weak correlation between them (S3 Fig). Moreover, under various cutoff values, there was no significant correlation between KYNU+macrophages and the overall survival of LUAD patients (Fig 3B). Then, all KYNU+macrophage LUAD samples were divided in two groups, including high KYNU expression and low expression LUAD groups, according to the median KYNU expression. The prognosis of LUAD patients in two groups showed no significant difference (Fig 3C).

To further estimate the potential impacts of KYNU on LUAD patients’ responses to immunotherapy, Tumor Immune Dysfunction and Exclusion (TIDE) analysis was employed to predict the KYNU+macrophages ratio in different patients. The results indicated that a significantly higher proportion of KYNU+macrophages was found in Responses LUAD patients, compared to Non-Response patients (Fig 3D).

**Fig 4:**
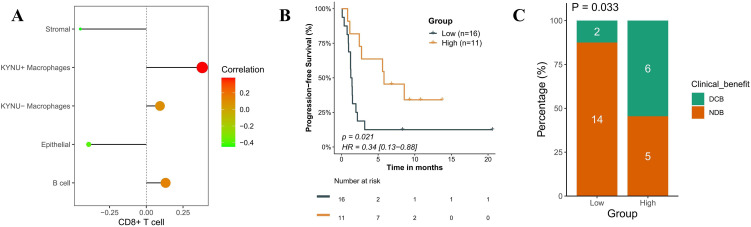
Clinical value of KYNU in immunotherapy responses of LUAD patients basing on GEO cohort. **(A)** The correlation analysis between CD8 + T cells and other cells. **(B)** Kaplan-Meier curve based on high and low KYNU expression LUAD groups. **(C)** The clinical benefit difference between LUAD patients with high and low proportions of KYNU+ macrophages. DCB: durable clinical benefit; NCB: no clinical benefit.

Additionally, the correlation between CD8 + T cells and other cells was also analyzed, using GSE135222. Among all cells, CD8 + T cells exhibited the strongest correlation with KYNU+macrophages (r = 0.38), whilst the weakest correlation with KYNU-macrophages (Fig 4A). Under various cutoff values, there was no significant correlation between KYNU-macrophages and the overall survival of LUAD patients (S4 Fig). Compared to lo w KYNU expression LUAD patients, KYNU highly expressed LUAD patients had significantly better progression-free survival (PFS) (Fig 4B). Moreover, in LUAD patients with higher proportions of KYNU+macrophages, significantly more patients could benefit from the immunotherapy (Fig 4C), implying the potential association between KYNU+macrophages and immunotherapy responses.

### KYNU related immune landscape in LUAD

**Fig 5:**
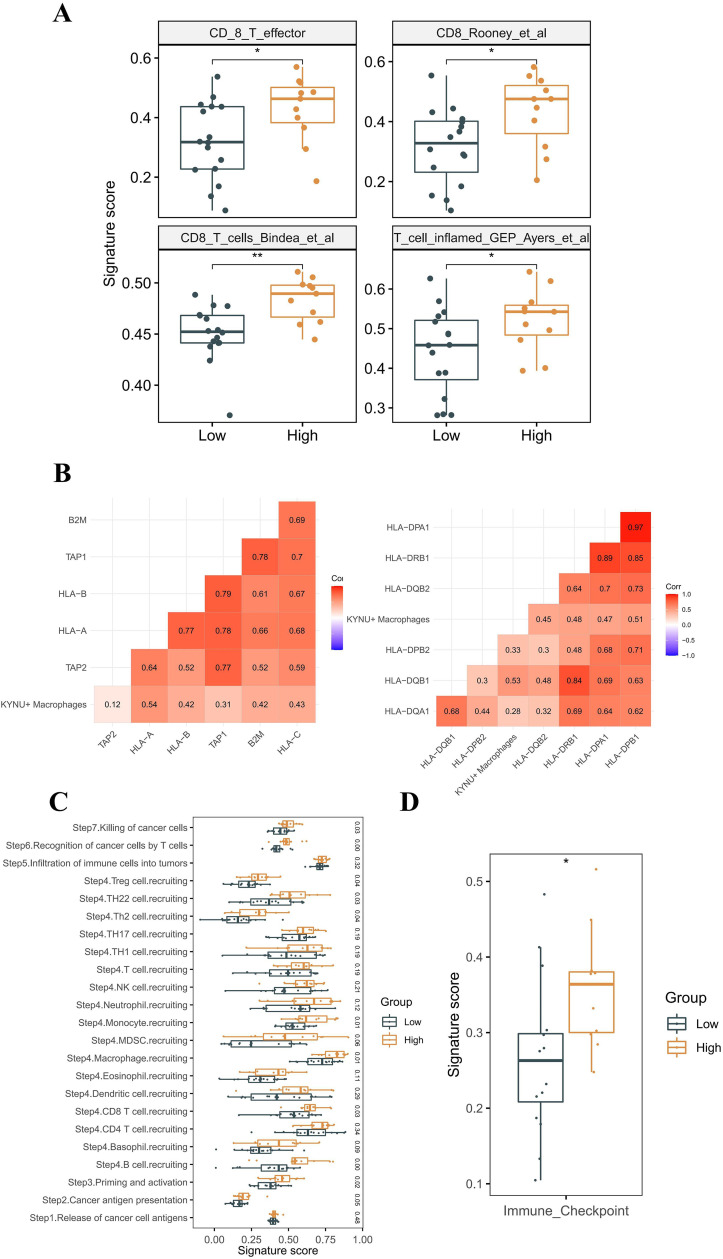
KYNU related immune landscape in LUAD. **(A)** T cell signature score between high and low KYNU+ macrophage groups. **(B)** The correlation between KYNU+ macrophages and MHC-I/ MHC-II molecules. Left: MHC-I molecules; Right: MHC-II molecules. **(C)** The cancer immunity cycle signature score between high and low KYNU+ macrophage groups. **(D)** The correlation between KYNU+ Macrophages and several crucial immune checkpoints.

Next, the KYNU related immune landscape was further investigated in LUAD, in order to obtain more information regarding the contribution of KYNU to TME. The ssGSEA was then used to analyze the T cell related signature between high and low KYNU+macrophage groups, the results suggested that a significantly higher T cell related signature score was found in high KYNU+macrophage group (Fig 5A). Moreover, we found a significantly high correlation between KYNU+macrophages and MHC-I/ MHC-II molecules (Fig 5B). Regarding the 7 steps in cancer immunity cycle, totally 10 types of signature scores were significantly higher in high KYNU+macrophage group, such as cancer antigen presentation, priming and activation, B cell recruiting, and CD8 T cell recruiting (Fig 5C). Besides, we have also analyzed the correlation between KYNU+ Macrophages and several crucial immune checkpoints. We found that the immune checkpoint signature score was significantly higher in high KYNU+macrophage group (Fig 5D, left), and KYNU+macrophages showed strong correlation with PDCD1LG2, HAVCR2, PDCD1, LAG3 (Fig 5D, right).

### Functional pathways between high and low KYNU+macrophage LUAD groups

**Fig 6:**
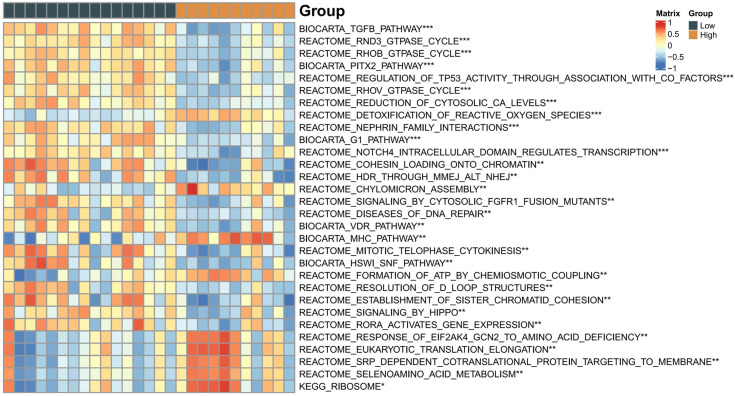
Functional pathways between high and low KYNU+ macrophage LUAD groups.

Finally, the distinct functional pathways were analyzed between high and low KYNU+macrophage groups employing GSVA algorithm. All significantly enriched pathways were displayed in Fig 6. In high KYNU+macrophage group, MHC_PATHWAY was significantly activated. The signature scores of TGFB_PATHWAY, HSWI_SNF_PATHWAY, and SIGNALING_BY_HIPPO were significantly higher in low KYNU+macrophage group (Fig 6). Our data indicated that there were indeed distinct functional pathway pattern between high and low KYNU+macrophage groups.

## Discussion

Due to the poor prognosis of LUAD patients, an increasing number of researchers have devoted significant efforts to understanding TIME [23], in order to further illustrate the underlying mechanisms and propose novel treatment strategies of LUAD. In our present study, KYNU exhibited extremely high expression in macrophages in LUAD, and KYNU expressed macrophages were indeed contributed to the distinct TIME feature of LUAD. Moreover, KYNU expressed macrophages were probably associated with the enhanced antigen presentation of LUAD patients.

Firstly, to investigate more details in TME of LUAD, we have analyzed the single cell RNA-seq data, and noticed that KYNU mainly expressed in Macrophages and Monocytes among all immune cells in LUAD. In normal cells, it has been indicated that KYNU participated in the biosynthesis of NAD + via kynurenine pathway, meanwhile aberrant KYNU expression involved in metabolic diseases [24,25]. Whereas, KYNU related reports focused on its role in cancer were limited. In breast cancer, KYNU has been evidenced as a potential target of CD44, which functioned together and activated the key PI3K/AKT pathway [17]. Moreover, in colorectal cancer, KYNU probably involved in the tumor promoting role of TDO2 through TDO2-KYNU-AhR pathway [26]. However, our present work has focused on the role of KYNU in macrophages in LUAD for the first time. After functional enrichment analysis, we found that the upregulated DEGs in KYNU+macrophages were predominantly enriched in cytokine production, T cell proliferation, and antigen processing and presentation related GO terms, implying a relatively activated immune status in KYNU+macrophage LUAD samples. Moreover, the strongly correlated CD8 + T cells and significantly higher T cell related signature score in KYNU+macrophage LUAD samples further supported our hypothesis. In TIME, the immune cells are able to mediate adaptive immune responses, among which CD8 + T cells have been widely known as antitumor effector cells, exerting cytotoxic roles [27,28]. The infiltration and activation of CD8 + T cells are obviously critical for establishing antitumor immunity. Harden et al. have demonstrated a positive correlation between KYNU expression and disease severity as well as inflammation, besides they have also indicated that the tryptophan metabolites downstream of KYNU probably increased some cytokines and chemokines in psoriasis [29]. Therefore, they illustrated that KYNU might serve as a switch between immunosuppressive versus inflammatory events [29]. Whereas, whether KYNU played a similar dual role in LUAD remained to be further explored in the future.

Subsequently, the cellular communication related results indicated a high correlation between KYNU+macrophages and Major Histocompatibility Complex (MHC)-I/ MHC-II molecules. MHC-class I (MHC-I) molecules were expressed by most nucleated cells, mainly presenting endogenously-derived peptide antigens to CD8 + T cells [30].Upon recognizing the tumor, CD8 + T cells can kill tumor cells through perforin- or FAS-dependent pathways while also damaging tumors by inducing inflammatory responses [31]. MHC-class II (MHC-II) molecules were expressed by professional antigen presenting cells (pAPCs) such as B cells and macrophages, primarily presenting exogenously-derived peptide antigens to CD4 + T cells [30]. Moreover, our GSVA results indicated that MHC pathway was significantly activated in high KYNU+macrophage group. It has been reported that tumor-specific MHC-II expression could elevated tumor recognition by the immune system, thereby improving patients’ responses to immune checkpoint inhibitor (ICI) and exerting key roles in immunotherapy [30,32]. In this regard, our findings indeed suggest that LUAD patients with a higher proportion of KYNU+ macrophages are more likely to benefit from immunotherapy. This could help refine precision treatment strategies for LUAD patients requiring immunotherapy in clinical practice. Nevertheless, the underlying mechanisms between KYNU and MHC pathway deserved further investigation in LUAD. Furthermore, this study still has limitations. For instance, it only employs a small sample dataset to preliminarily explore the potential of KYNU as a biomarker for immunotherapy. Subsequent research should validate the clinical value of KYNU by collecting real‑world treatment response data from large‑scale, prospective cohorts of patients undergoing immunotherapy, and by performing standardized ROC/AUC analyses as well as multivariable Cox regression analyses. Second, our conclusions are primarily derived from transcriptomic analysis of public databases. While these data are valuable, the specific biological functions of KYNU remain unknown. Therefore, future studies are urgently needed to validate, at the protein level, the association between KYNU expression and patient response to immune checkpoint inhibitors as well as survival outcomes. This should be conducted in independent, large-scale lung adenocarcinoma cohorts with comprehensive clinical follow-up and records of immunotherapy responses, utilizing techniques such as immunohistochemistry or immunofluorescence. Moreover, the specific anti-tumor and immunomodulatory functions of KYNU should be further investigated and validated in cellular or animal models.

## Conclusions

To summarize, our study has highlighted the role of KYNU in LUAD macrophages for the first time. After combining single-cell and bulk RNA sequencing data, we have demonstrated the potential promoting role of KYNU on distinct immune features of LUAD patients. KYNU expressed macrophages probably correlated with the enhanced antigen presentation of LUAD samples, which is also the basis of the clinical value of KYNU. Our present work has revealed more details of KYNU in macrophages and TME of LUAD, meanwhile KYNU exhibits important potential in serving as a target of immunotherapy, deserving further exploration.

## Supporting information

S1 Fig The expression pattern of KYNU in LUAD samples at a single cell resolution. (A) All 9 cell clusters annotated in GSE123902. (B-C) KYNU was mainly expressed in Monocytes and Macrophages in GSE123902.
(JPG)

S2 Fig KYNU expression was correlated with macrophages and enhanced antigen presentation in LUAD. (A) The differentially expressed genes between KYNU+ macrophages group and KYNU-macrophages group. (B-C) The GO enrichment results of upregulated genes in KYNU+ macrophages group (B) and KYNU-macrophages group (C), respectively. (D) The cell communications among KYNU+ macrophages, KYNU-macrophages, and other cells. (E) The interaction network involving MHC-I signaling pathway and MHC-II signaling pathway between KYNU+macrophages and KYNU-macrophages. (F) Correlation between MHC-I/ MHC-II molecules and KYNU+ macrophages, KYNU-macrophages.
(JPG)

S3 Fig The correlation between KYNU and the infiltration proportions of Tumor purity, B cell, CD8 + T cells, CD4 + T cells, Macrophage, Neutrophil, Dendritic Cell. (JPG)

S4 Fig The prognostic value of KYNU-macrophage under various cutoff values. (JPG)

## Abbreviations

NSCLC: Non-small cell lung cancer
LUAD: Lung adenocarcinoma
TKI: Tyrosine kinase inhibitor
TIME: Tumor immune microenvironment
TME: Tumor microenvironment
KYNU: Kynureninase
TAM: Tumor‑associated macrophage
UMI: Unique molecular identifier
HVGs: Highly variable genes
PCA: Principal component analysis
UMAP: Uniform manifold approximation and projection
CI: Confidence interval
TCGA: The Cancer Genome Atlas
GEO: Gene Expression Omnibus
GSVA: Gene set variation analysis
TIDE: Tumor immune dysfunction and exclusion
DEGs: Expressed genes
PFS: progression-free survival
MHC-I: Major Histocompatibility Complex-class I
MHC-II: Major Histocompatibility Complex-class II
ICI: Immune checkpoint inhibitor
