# Supplementary figures and images for: KYNU in macrophages contributes to the unique immune feature of LUAD via integrating single-cell and bulk RNA sequencing data: an exploratory analysis
Source: PLoS One. 2026 Jun 12;21(6):e0351622. doi: 10.1371/journal.pone.0351622 (PMC13262878; doi:10.1371/journal.pone.0351622)

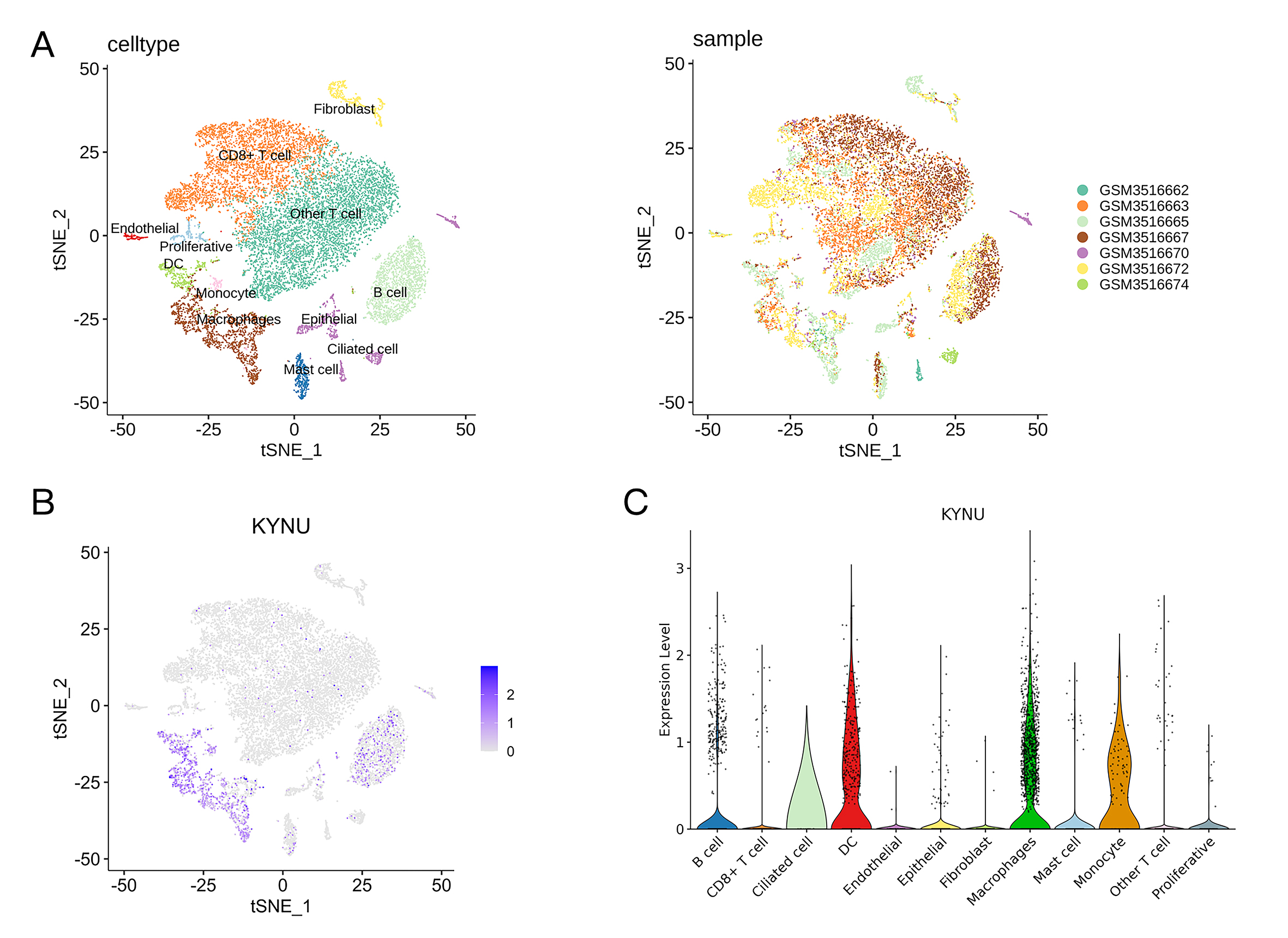

Supplement: S1 Fig — (A) All 9 cell clusters annotated in GSE123902. (B-C) KYNU was mainly expressed in Monocytes and Macrophages in GSE123902. (JPG) [file pone.0351622.s001.jpg]

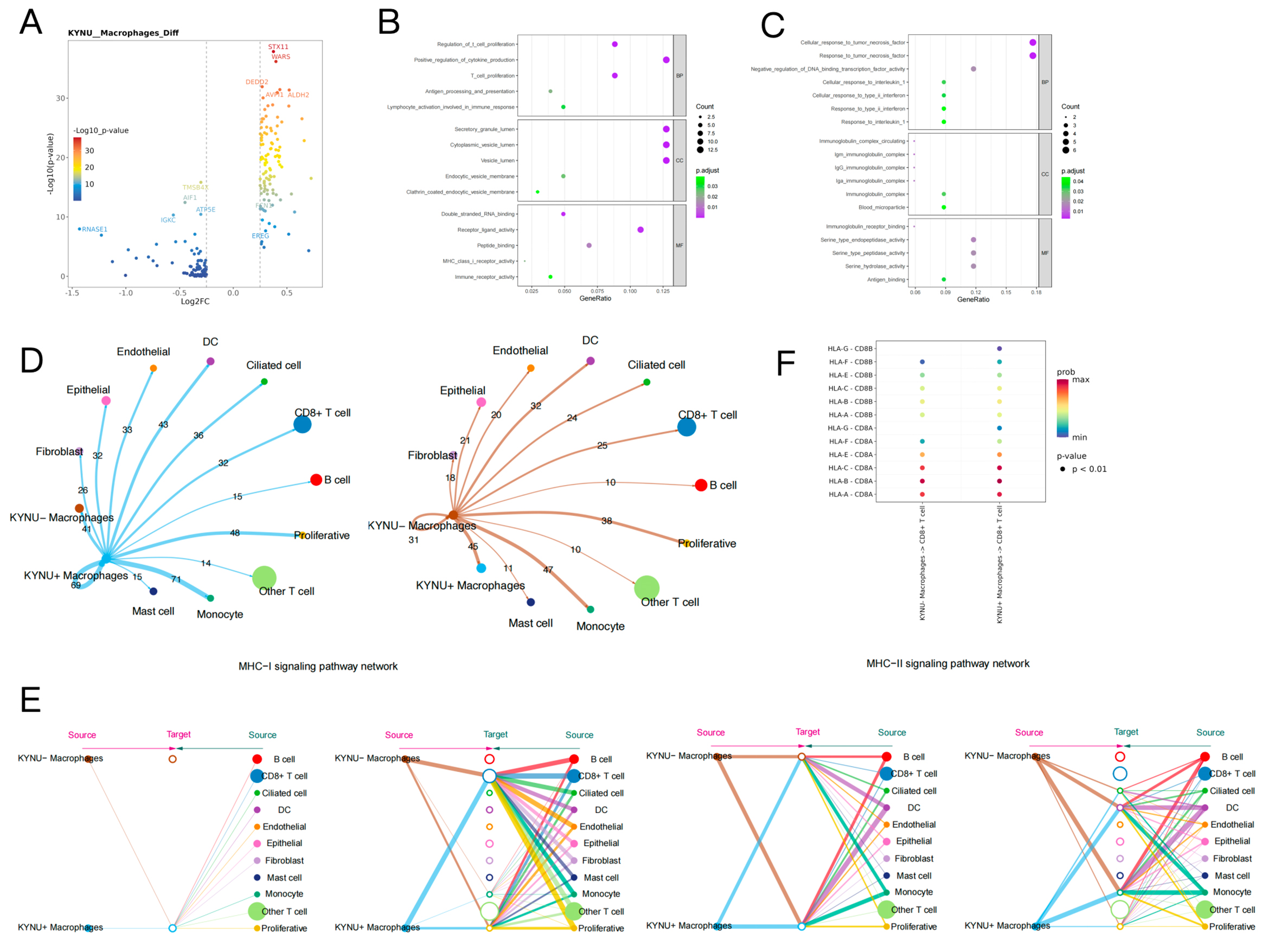

Supplement: S2 Fig — (A) The differentially expressed genes between KYNU+ macrophages group and KYNU-macrophages group. (B-C) The GO enrichment results of upregulated genes in KYNU+ macrophages group (B) and KYNU-macrophages group (C), respectively. (D) The cell communications among KYNU+ macrophages, KYNU-macrophages, and other cells. (E) The interaction network involving MHC-I signaling pathway and MHC-II signaling pathway between KYNU+macrophages and KYNU-macrophages. (F) Correlation between MHC-I/ MHC-II molecules and KYNU+ macrophages, KYNU-macrophages. (JPG) [file pone.0351622.s002.jpg]

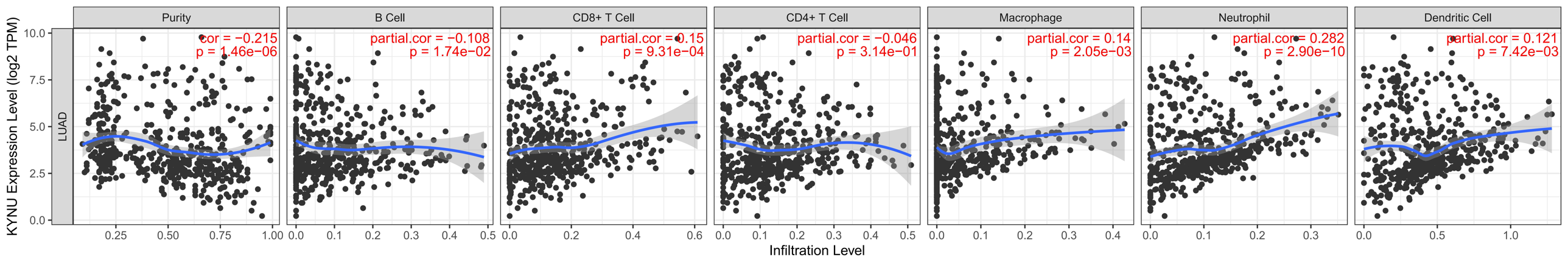

Supplement: S3 Fig — (JPG) [file pone.0351622.s003.jpg]

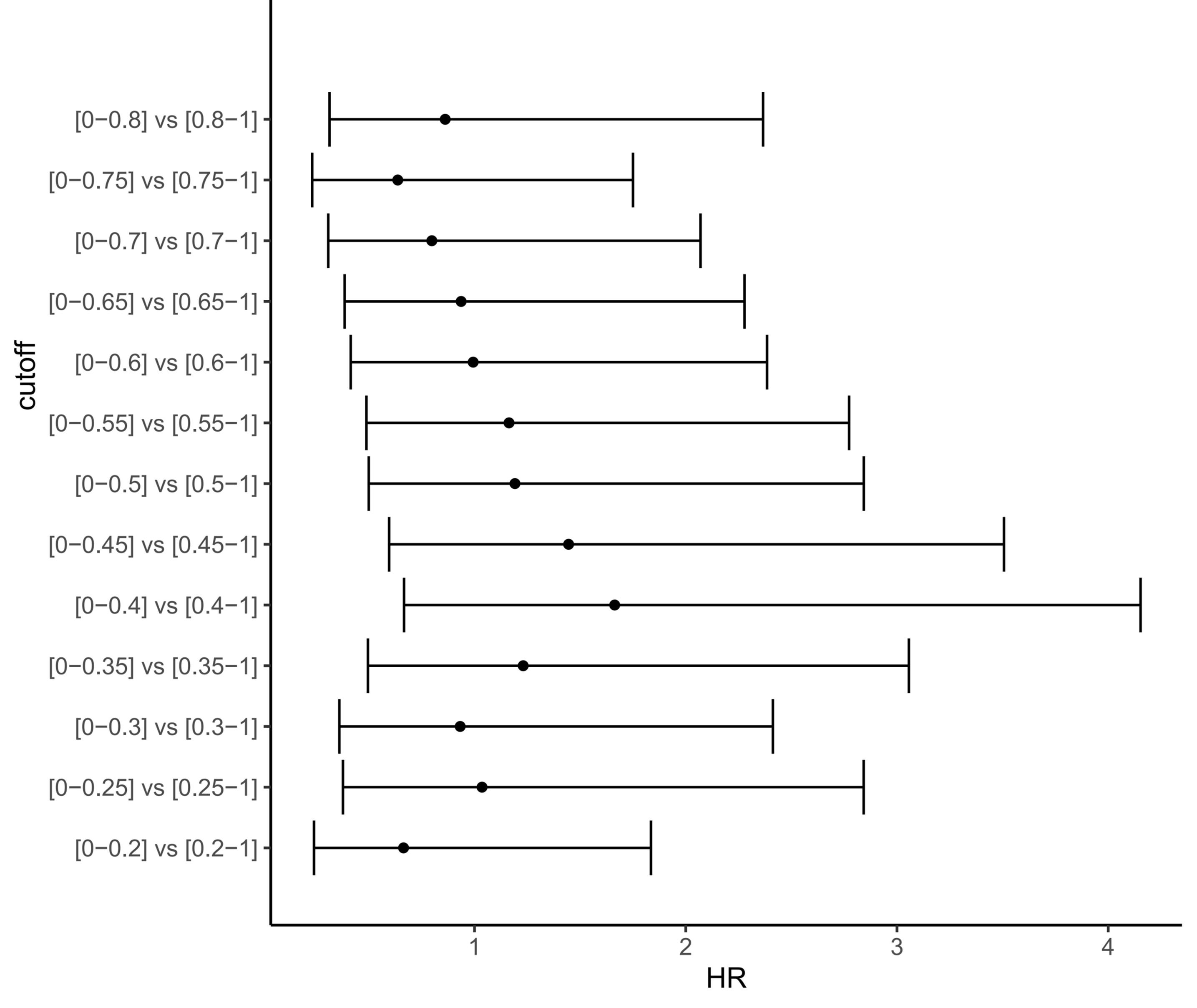

Supplement: S4 Fig — (JPG) [file pone.0351622.s004.jpg]
